# Radiation Dose Reduction for Coronary Artery Calcium Scoring Using a Virtual Noniodine Algorithm on Photon-Counting Detector Computed-Tomography Phantom Data

**DOI:** 10.3390/diagnostics13091540

**Published:** 2023-04-25

**Authors:** Nicola Fink, Emese Zsarnoczay, U. Joseph Schoepf, Jim O’Doherty, Joseph P. Griffith, Daniel Pinos, Christian Tesche, Jens Ricke, Martin J. Willemink, Akos Varga-Szemes, Tilman Emrich

**Affiliations:** 1 Division of Cardiovascular Imaging, Department of Radiology and Radiological Science, Medical University of South Carolina, 25 Courtenay Dr, Charleston, SC 29425, USA; 2 Department of Radiology, University Hospital, LMU Munich, Marchioninistr. 15, 81377 Munich, Germany; 3 Medical Imaging Center, Semmelweis University, Korányi Sándor utca 2, 1083 Budapest, Hungary; 4 Siemens Medical Solutions, 40 Liberty Boulevard, Malvern, PA 19355, USA; 5 Department of Cardiology, Munich University Clinic, Ludwig-Maximilians-University, Marchioninistr. 15, 81377 Munich, Germany; 6 Department of Radiology, Stanford University School of Medicine, 291 Campus Drive, Stanford, CA 94305, USA; 7 Department of Diagnostic and Interventional Radiology, University Medical Center of Johannes-Gutenberg-University, Langenbeckstr. 1, 55131 Mainz, Germany; 8 German Centre for Cardiovascular Research, Partner Site Rhine-Main, 55131 Mainz, Germany

**Keywords:** coronary artery disease, photon-counting detector, computed tomography, calcium scoring, virtual noniodine, radiation dose

## Abstract

**Background:** On the basis of the hypothesis that virtual noniodine (VNI)-based coronary artery calcium scoring (CACS) is feasible at reduced radiation doses, this study assesses the impact of radiation dose reduction on the accuracy of this VNI algorithm on a photon-counting detector (PCD)-CT. **Methods:** In a systematic in vitro setting, a phantom for CACS simulating three chest sizes was scanned on a clinical PCD-CT. The standard radiation dose was chosen at volumetric CT dose indices (CTDI_Vol_) of 1.5, 3.3, 7.0 mGy for small, medium-sized, and large phantoms, and was gradually reduced by adjusting the tube current resulting in 100, 75, 50, and 25%, respectively. VNI images were reconstructed at 55 keV, quantum iterative reconstruction (QIR)1, and at 60 keV/QIR4, and evaluated regarding image quality (image noise (IN), contrast-to-noise ratio (CNR)), and CACS. All VNI results were compared to true noncontrast (TNC)-based CACS at 70 keV and standard radiation dose (reference). **Results:** IN_TNC_ was significantly higher than IN_VNI_, and IN_VNI_ at 55 keV/QIR1 higher than at 60 keV/QIR4 (100% dose: 16.7 ± 1.9 vs. 12.8 ± 1.7 vs. 7.7 ± 0.9; *p* < 0.001 for every radiation dose). CNR_TNC_ was higher than CNR_VNI_, but it was better to use 60 keV/QIR4 (*p* < 0.001). CACS_VNI_ showed strong correlation and agreement at every radiation dose (*p* < 0.001, r > 0.9, intraclass correlation coefficient > 0.9). The coefficients of the variation in root-mean squared error were less than 10% and thus clinically nonrelevant for the CACS_VNI_ of every radiation dose. **Conclusion:** This phantom study suggests that CACS_VNI_ is feasible on PCD-CT, even at reduced radiation dose while maintaining image quality and CACS accuracy.

## 1. Introduction

Coronary artery calcium scoring (CACS) is a strong predictor of future adverse cardiovascular events and outcomes [1,2,3,4,5], and is thereby used for cardiovascular risk stratification [6,7]. The cardiac computed tomography (CT)-based Agatston method (tube voltage, 120 kVp; reconstruction with slice thickness, 3.0 mm) with a threshold of 130 Hounsfield units (HU) is considered the standard method for CACS [8]. The 2019 American College of Cardiology/American Heart Association (ACC/AHA) Guideline on the Primary Prevention of Cardiovascular Disease supports the assessment of CACS for further decision making in patients at intermediate risk and selected patients at borderline risk [9]. However, when considering this method as a screening tool to prevent adverse cardiovascular events, the radiation dose remains a concern. 

Photon-counting detector (PCD)-CT, a recent and promising technology, enables energy discrimination at high spatial and temporal resolutions by separately counting every incoming photon, weighing it with regard to its energy [10,11,12,13], and allowing for low-dose imaging with better image quality than that of energy-integrating detectors (EIDs) [14,15,16]. Moreover, compared to dual-energy CT systems, improved spectral imaging enables multimaterial differentiation [12], e.g., among soft tissue, calcium, and iodine. By creating virtual noniodine (VNI) images on the basis of coronary CT angiography (CCTA) data, the true noncontrast (TNC) examination normally performed for CACS could potentially be eliminated, possibly reducing both the examination time and total radiation burden to the patient.

In this context, an iodine-removal algorithm specifically developed for PCD-CT outperformed virtual noncontrast (VNC) reconstructions for CACS at a standard radiation dose [17]. Due to the advantages of PCD-CT and accordingly this new VNI algorithm, we hypothesized that VNI reconstructions enable accurate CACS even in low-dose examinations. 

Therefore, this study assesses the impact of radiation dose reduction on image quality and CACS accuracy using a VNI algorithm on PCD-CT phantom data. In a systematic analysis of a well-standardized chest phantom with a CACS insert and two extension rings simulating three different chest diameters, VNI-based CACS was examined at four different radiation dose levels regarding the calcium score and image quality. 

## 2. Materials and Methods

### 2.1. Phantom Setup

For this study, a commercially available anthropomorphic chest phantom (QRM GmbH, Moehrendorf, Germany) was used with an insert designed for calcium scoring at the heart position. This insert contained nine cylinders with different diameters (1, 3, and 5 mm) and different densities (200, 400, and 800 mg/cm^3^ calcium hydroxyapatite), and two calibration rods (one water-equivalent and one with 200 mg/cm^3^ calcium hydroxyapatite) as recently described [17]. To simulate the different chest diameters, the phantom was scanned with and without extension rings, resulting in a total of three chest diameters: 300 × 200 mm (small; no extension ring), 350 × 250 mm (medium; extension ring M), and 400 × 300 mm (large; extension ring L).

### 2.2. Data Acquisition

All phantoms were scanned using a first-generation dual-source PCD-CT (NAEOTOM Alpha; Siemens Healthineers, Forchheim, Germany) containing two PCDs (cadmium telluride; collimation of 144 × 0.4 mm). The tube voltage was 120 kVp in QuantumPlus scan mode, gantry rotation time was 0.25 s, and scans were performed with a simulated electrocardiogram (ECG) signal at 60 beats per minute (bpm) and an ECG triggering with a single phase of 75%. As previously described [18], the standard radiation dose (100%) was chosen according to clinically used protocols with volumetric CT dose index (CTDI_Vol_) values of 1.5, 3.3, and 7.0 mGy for the small, medium-sized, and large phantoms, respectively [19,20,21], with calculated mean CTDI_Vol_ of 1.4 ± 0.1, 3.1 ± 0.04, and 6.1 ± 0.2 mGy, respectively. Mean CTDI_Vol_ values were calculated with the CT scanner and extracted from patient exposure protocol data. Radiation dose was gradually reduced by adjusting tube current modulation, which resulted in four different dose levels: 100% (standard radiation dose), 75%, 50%, and 25%. Detailed radiation dose parameters are illustrated in Table 1. 

Since interscan variability may be high in CACS [22], each scan was repeated five times with the slight repositioning of the phantom between the scans (approximately 2 mm translational and 2 degrees rotational) [23].

### 2.3. Image Reconstruction

Reconstructions were performed using a proprietary offline raw data image reconstruction platform (ReconCT version 15.0.57554.0; Siemens Healthineers) with the following parameters: reconstruction kernel Qr36f, slice thickness 3.0 mm, slice increment 1.5 mm, as recommended by the vendor, and field of view 200 mm. In addition to the standard options, such as slice thickness and convolutional kernel, PCD-CT offers further possibilities in image reconstruction. Measuring the energy of every incoming photon separately enables to create virtual monoenergetic images (VMI) instead of conventional polychromatic CT reconstructions [10]. In addition, iterative reconstructions (IR) can be used to substantially lower image noise [24,25], which is particularly advantageous in low-dose imaging [26,27,28,29]. Since conventional IR algorithms cannot simply be transferred to PCD-CT systems [30], the vendor of this first-generation PCD-CT introduced a quantum IR (QIR) algorithm with four strength levels. The images of all four radiation dose levels were postprocessed using a VNI algorithm (PureCalcium, Siemens Healthineers) as previously described [17], and reconstructed at two different VMI and QIR levels: 55 keV/QIR1 and 60 keV/QIR4. These reconstructions showed that VNI-based scores were the closest to the corresponding scores from reference TNC scans [31]. TNC reconstructions without VNI postprocessing at 70 keV and QIR off at the standard radiation dose served as the reference as recommended by the vendor (given by the factory protocol).

### 2.4. Image Analysis

Scans were analyzed by a radiologist with four years of experience in cardiovascular imaging regarding image quality and CACS. Image quality was determined on the basis of image noise (IN) and the contrast-to-noise ratio (CNR). Regions of interest (ROIs) of 1.5 cm^2^ were used to evaluate the consistency of the CT numbers (HU, mean, and standard deviation (SD)) in the center of the calibration rod and the background of the calcium scoring insert of the phantom. Each measurement was performed three times at different locations, and the mean values were calculated for further analysis. IN was defined as the SD of the background measurements, and the CNR was calculated according to the following formula [32]:

$$CNR=\frac{HU_{Calcium\;Calibration\;Rod}-HU_{Phantom\;Background}}{SD_{Phantom\;Background}}$$

According to the guideline for minimizing radiation exposure during the acquisition of coronary artery calcium scans with the use of multidetector computed tomography [33], the recommended IN targets of 20 HU for small/medium-sized phantoms and 23 HU for large phantoms were used to further compare the IN between different reconstructions. CACS quantification was performed on VNI (CACS_VNI_) and TNC (CACS_TNC_) datasets using commercially available software (syngo.via Version VB60; Siemens Healthineers) that highlights calcified lesions by discriminating them from the background using a threshold of 130 HU [8], as recently described [17].

### 2.5. Statistical Analysis

Statistical analysis was performed using GraphPad Prism (Version 8.4.2; GraphPad, San Diego, CA, USA) and MedCalc (Version 19.4; MedCalc, Ostend, Belgium). Continuous variables were tested for normality using the Kolmogorov–Smirnov test. Normally distributed values are reported as mean ± standard deviation, and non-normally distributed values as the median with an interquartile range. Categorical values are reported as absolute frequencies and proportions. Depending on the distribution and number of variables, we used the paired-samples t-test or Wilcoxon matched-pairs signed rank test and one-way ANOVA with the Greenhouse correction or Friedman test to evaluate differences among the different reconstructions (VNI postprocessed, 55 keV/QIR1; VNI postprocessed, 60 keV/QIR4; and without VNI postprocessing, 70 keV/QIR off), and between among radiation dose levels (100%, 75%, 50% and 25%). The Bonferroni correction was applied for multiple t tests, and a *p* value of 0.05/3 = 0.017 was considered significant for different reconstructions and 0.05/6 = 0.008 for different radiation dose levels. The correlation between the CACS_VNI_ of every radiation dose level and the CACS_TNC_ of standard radiation dose was assessed using the Pearson or Spearman correlation coefficient (r) depending on the distribution. The corresponding agreement was tested using Bland–Altman analysis (mean bias, upper and lower limits of agreement (LoA)) and single-rater intraclass correlation (ICC) with 2-way mixed effects and absolute agreement (<0.5: poor; 0.5 to 0.75: moderate; 0.75 to 0.9: good; >0.9: excellent [34]). The percentage deviation of IN, CNR, and CACS between VNI and TNC reconstructions was calculated as follows:

$$Percentage\;Deviation=\frac{IN/CNR/CACS_{VNI}-IN/CNR/CACS_{TNC}}{IN/CNR/CACS_{TNC}}\times 100\%$$

The coefficients of variation (CV) of the root-mean squared error (RMSE) were calculated to evaluate the variation between CACS_VNI_ at different radiation dose levels and CACS_TNC_ at the standard radiation dose. Values < 10% were considered acceptable agreement and thus clinically nonrelevant deviation [35].

## 3. Results

### 3.1. Image Quality

For every radiation dose level, mean IN_VNI_ was significantly lower than IN_TNC_ (*p* < 0.001 for all), and higher in VNI reconstructions at 55 keV/QIR1 compared to 60 keV/QIR4 (*p* < 0.001 for all) (Table 2).

**Figure 1:**
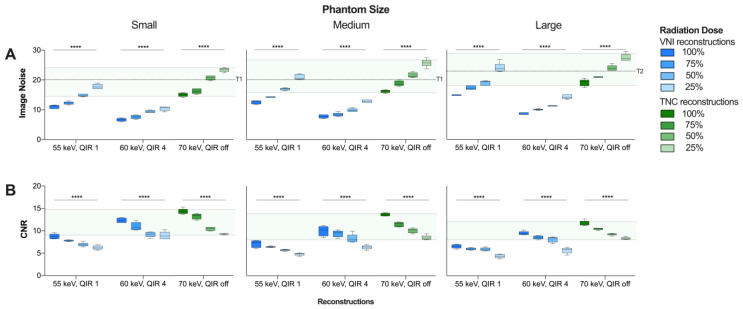
Comparison of (**A**) image noise levels (SD background) and (**B**) contrast-to-noise ratio between VNI (55 keV, QIR 1 and 60 keV, QIR 4, respectively) and TNC (70 keV, QIR off) reconstructions. Range of IN_TNC_ and CNR_TNC_ at dose level 100 to 25% highlighted in green. Recommended Image Noise Target: 20 HU in small/medium phantoms (T1) and 23 HU in large phantoms (T2), respectively [33]. **** *p* < 0.0001. CNR = contrast-to-noise ratio; HU = Hounsfield units; IN = image noise; QIR = quantum iterative reconstruction; SD = standard deviation; VNI = virtual noniodine.

The mean IN_VNI_ and IN_TNC_ increased significantly with decreasing radiation dose level from 100 to 25% (*p* < 0.001 for all) (Figure 1A). Compared to TNC reconstructions, IN was reduced by 23.5% and 54.0% at the standard radiation dose, and by 17.9% and 50.8% at the 25% radiation dose level using VNI reconstructions at 55 keV/QIR1 and 60 keV/QIR4, respectively.

The mean CNR_VNI_ was significantly lower than CNR_TNC_ (*p* < 0.001 for all), and lower in VNI reconstructions at 55 keV/QIR1 compared to 60 keV/QIR4 (*p* < 0.001 for all) (Table 2). Mean CNR_VNI_ and CNR_TNC_ decreased significantly with decreasing radiation dose level from 100 to 25% (*p* < 0.001 for all) (Figure 1B). In comparison to TNC reconstructions, CNR was reduced by 43.4% and 19.8% at standard radiation dose, and by 40.8% and 19.8% at radiation dose level 25% VNI reconstructions at 55 keV/QIR1 and 60 keV/QIR4, respectively.

### 3.2. Calcium Scoring

CACS_VNI_ at 55 keV/QIR1 and 60 keV/QIR4 of every radiation dose level from 100 to 25% showed strong correlation and excellent agreement with CACS_TNC_ at standard radiation dose (*p* < 0.001, r ≥ 0.88, ICC > 0.9 for all) (Table 3).

Compared to CACS_TNC_ at the standard radiation dose, CACS_VNI_ was significantly lower only at 55 keV/QIR1 and the 50% radiation dose level (625.8 ± 24.4 vs. 597.4 ± 40.0; *p* < 0.05), but there were no significant differences between CACS_TNC_ and CACS_VNI_ at any other reconstruction or radiation dose level. Within VNI reconstructions, scores at the 50 and 25% dose levels were significantly lower only at 55 keV/QIR1 compared to 60 keV/QIR4 (55 keV/QIR1: 597.4 ± 39.6 and 599.0 ± 58.4 vs. 60 keV/QIR4: 602.2 ± 38.2 and 611.5 ± 57.3, *p* < 0.05 and *p* < 0.01, respectively).

**Figure 2:**
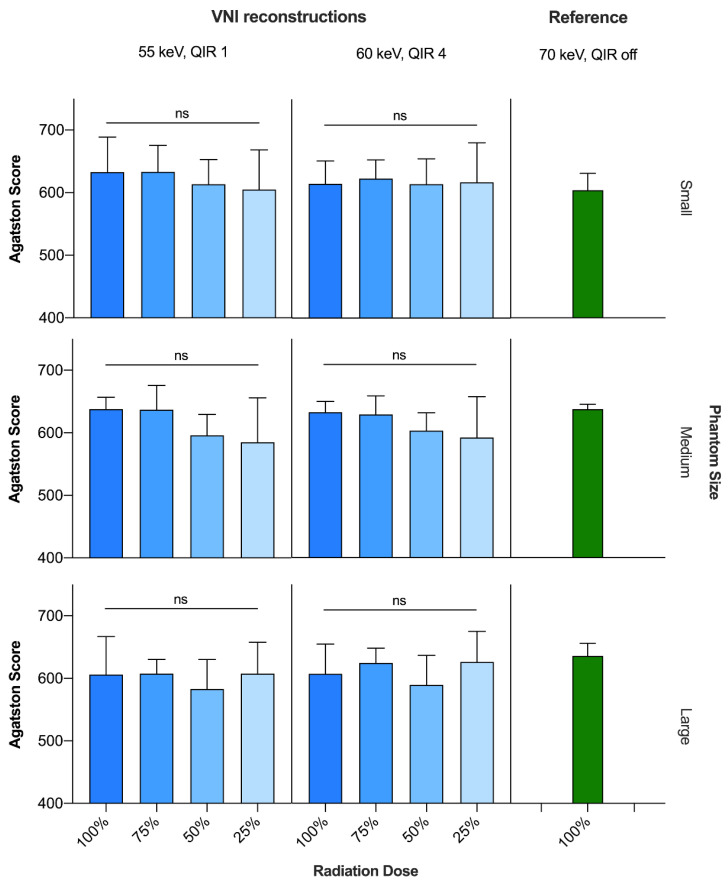
Comparison of Agatston scores between CACS_VNI_ (55 keV, QIR 1 and 60 keV, QIR 4, respectively) at different radiation dose levels and CACS_TNC_ (70 keV, QIR off) at standard radiation dose. ns = not significant; QIR = quantum iterative reconstruction; TNC = true noncontrast; VNI = virtual noniodine.

For the VNI reconstructions at 55 keV/QIR1 and at 60 keV/QIR4, there were no significant differences in calcium scores among the different radiation dose levels from 100 to 25% (Figure 2).

Averaged over all phantom sizes, total CACS_VNI_ scores deviated from CACS_TNC_ at standard radiation dose, by up to 4.5% and 3.7% for 55 keV/QIR1 and 60 keV/QIR4, respectively, each with the highest deviation at the 50% radiation dose level. Depending on the radiation dose, CV(RSME) values were 6.9% at 100%, 5.2% at 75%, 6.6% at 50% and 7.8% at the 25% radiation dose level when comparing the total CACS_VNI_ scores at 55 keV/QIR1 with CACS_TNC_ at standard radiation dose. The respective values of CACS_VNI_ at 60 keV/QIR4 were slightly lower: 5.4%, 4.0%, 6.2% and 7.1% (Table 3).

## 4. Discussion

This study investigated the impact of radiation dose reduction on image quality and CACS using a VNI algorithm on PCD data. The main findings of our study are as follows: First, VNI reconstructions at 55 keV/QIR1 and 60 keV/QIR 4 showed comparable or even better image quality in terms of noise than that of TNC reconstructions at 70 keV/QIR off. Second, CACS_VNI_ at every dose level from 100 to 25% showed excellent correlation and agreement with CACS_TNC_ at standard radiation dose with a clinically nonrelevant deviation of scores. Third, CACS_VNI_ at 60 keV/QIR4 seems to slightly outperform 55 keV/QIR1 in terms of image quality and score deviation.

Several studies demonstrated the feasibility of radiation dose reduced CACS without compromising image quality and scoring in TNC scans obtained by previous dual-source [20,33,36,37,38,39], as well as PCD CT [40,41,42,43]. However, due to its added diagnostic and prognostic value [44,45], CCTA and CACS are often combined in clinical practice; the latter are usually based on a separate TNC scan because of the difficulty in discriminating between iodine and calcium. The in 2022 updated version of the Coronary Artery Disease-Reporting and Data System (CAD-RADS 2.0) also supports the combination of CACS with CCTA: Due to the growing evidence that the overall plaque burden is prognostically relevant, a category “P” was added to the previous CCTA-based stenosis assessment to classify the plaque burden, ideally using the calcium score [46]. In this context, eliminating the need for a TNC scan by generating VNI images had been discussed as a method to reduce radiation dose exposure [47]. However, reducing radiation dose leads to increased image noise and artifacts, which may affect CACS by increasing noise in voxels that exceed the 130 HU threshold, thus are erroneously counted as calcified lesions. This interaction of radiation dose and image quality has to be considered, and, in accordance with the ALARA principle, radiation dose should not be as low as possible but as low as reasonably achievable.

In this study, the investigated VNI reconstructions showed similar or, regarding IN, even better image quality compared to that of TNC reconstructions. Regardless of radiation dose level, IN_VNI_ at 55 kev/QIR1 was reduced by around 20% in comparison to IN_TNC_, at 60 keV/QIR4 even by over 50%. Moreover, even at radiation dose of 25%, IN_VNI_ with 60 keV/QIR was lower than IN_TNC_ at standard radiation dose of 100%. Overall, CNR_VNI_ was significantly lower than CNR_TNC_ but less reduced at 60 keV/QIR4 than at 55 keV/QIR1. Overall, these results demonstrate improved image noise of VNI reconstructions, but at the same time lower CNR in comparison to TNC reconstructions with a slightly better performance of VNI at 60 keV/QIR4 compared to 55 keV/QIR1. Although lower CNR did not seem to significantly influence CACS values, it limits radiation dose reduction in VNI-based CACS. Furthermore, it underlines the importance of choosing appropriate VMI and QIR settings when reducing radiation dose. Better image noise of VNI reconstructions, even at low dose acquisitions, is likely due to the application of the QIR algorithm, which also explains slightly better performance of 60 keV reconstructions with QIR 4 compared to 55 keV with QIR 1. Iterative reconstructions reduce image noise [24] and allow for low dose calcium scoring [26,28]. At the same time, reducing noise and blooming artifacts using iterative reconstructions may affect CACS by resulting in fewer voxels exceeding the 130 HU threshold and thus decreasing calcium scores [48,49,50,51].

Despite the application of iterative reconstructions, this study demonstrated strong correlation and excellent agreement of CACS_VNI_ with CACS_TNC_ at every radiation dose level from 100 to 25%. This is in line with a previous study that evaluated the performance of the same VNI algorithm at standard radiation dose and demonstrated high accuracy for CACS [17]. CACS_VNI_ differed significantly from CACS_TNC_ at standard radiation dose only at 55 keV/QIR1 and dose level 50% but showed no significant differences at any radiation dose level for VNI reconstructions at 60 keV/QIR4. At the same time, at radiation doses 50 and 25%, CACS_VNI_ was significantly higher at 60 keV/QIR4 than the respective values at 55 keV/QIR1. Eberhard et al. recently evaluated the effects of different VMI and QIR levels on CACS using TNC PCD-CT scans and demonstrated increasing scores with increasing keV as well as decreasing QIR level, without significant differences among various radiation dose levels [52]. According to these results, scores should be higher at 55 keV/QIR1 than that at 60 keV/QIR4. However, while Eberhard et al. evaluated CACS using TNC reconstructions, these effects may differ due to the use of the VNI algorithm in this study. Furthermore, VNI reconstructions at 50 keV/QIR1 seem to be more susceptible to the influence of low radiation dose levels. The exact reason for this effect remains unclear but might be due to the lower QIR level.

However, overall and for both postprocessing settings, CACS_VNI_ values maintained accurate and stable, even when decreasing the radiation dose to 25%, without any significant differences between different radiation dose levels. Regardless of radiation dose level, CACS_VNI_ values differed less than 5% from CACS_TNC_ at standard radiation dose. At the same time, CV(RSME) values of CACS_VNI_ were less than 10% at each investigated dose level, demonstrating a clinically nonrelevant variation compared to CACS_TNC_ at standard radiation dose. Regarding CACS deviation and variation, VNI reconstructions at 60 keV/QIR4 performed slightly better than those at 55 keV/QIR1.

Radiation reduction is important since CACS is a strong predictor of cardiovascular morbidity and mortality in symptomatic [53] and, more importantly, asymptomatic individuals [4,54]. In addition to the calcium score, plaque density also determines the individual cardiovascular risk, with lower risk in case of high-density plaques [55,56]. The 2010 ACC/AHA Guideline for Assessment of Cardiovascular Risk in Asymptomatic Adults suggests that CACS is reasonable to consider in asymptomatic patients at intermediate risk and possibly even at low to intermediate risk [57]. However, radiation exposure was one reason why CACS was downgraded from a Class IIA to a Class IIB recommendation for patients for whom risk-based treatment decision remains uncertain after formal risk stratification in the 2013 ACC/AHA Guideline on the Assessment of Cardiovascular Risk [58]. Hence, it is still handled as such in the 2019 ACC/AHA Guideline on the Primary Prevention of Cardiovascular Disease [9]. The benefit of CACS screening has to be weighed against the risk of exposing asymptomatic patients to ionizing radiation [57,59,60] and strategies have to be developed to keep radiation dose as low as reasonably achievable while maintaining image quality and CACS values. To our knowledge, this is the first study to evaluate the feasibility of radiation dose reduced CACS using a VNI reconstruction algorithm on PCD data and indicates its feasible implementation in clinical practice to ensure appropriate individual cardiac risk stratification using CACS. This enables adding the prognostic value of CACS to every CCTA examination, thus providing the full spectrum of CT-based cardiac assessment while addressing concerns about radiation exposure.

Our study has the following limitations: First, our results were based on a static phantom that only contains calcium but no iodine inserts, which means that we mainly investigated the performance of this VNI reconstruction algorithm in not erroneously removing calcium. However, this systematic phantom study is crucial as a basis for further in vivo studies to analyze whether a reduction in radiation dose is possible, and to prevent patients from being exposed to radiation for scans that may not be diagnostic. Second, we only investigated four predefined radiation dose levels without investigating the lowest possible level. Third, the radiation dose levels examined in this study were lower than those usually expected in a CCTA scan. Fourth, this study did not investigate the impact of different plaque characteristics, such as diameter and density, on VNI-based CACS. These characteristics determine individual cardiovascular risk [55,56], so this evaluation should be part of future studies. Nevertheless, the results of this study provide the first standardized overview of the accuracy of low-dose CACS using VNI reconstructions. Further in vivo or ex vivo studies should be conducted to validate these results in order to transfer them to clinical practice. These studies should then preferably also investigate the impact of cardiac motion on the VNI algorithm’s performance.

## 5. Conclusions

In conclusion, the present study indicated the feasibility of low-dose CACS without decisively compromising image quality using a VNI reconstruction algorithm. However, lower CNR values compared to TNC-based CACS have to be taken into account in VNI-based CACS. Although the CACS results of the present study suggest that this does not significantly impact calcium scores, due to the slightly better image quality performance and less score deviation, VNI reconstructions at 60 keV, QIR 4 should be preferred over those at 55 keV, QIR 1 when reducing the radiation dose. This may enable a radiation dose reduction in VNI-based CACS of potentially up to 75%.

## Figures and Tables

**Table 1:** Calculated image acquisition parameters at different radiation dose levels.

	Phantom Size	Radiation Dose Level			
		100%	75%	50%	25%
CTDI_Vol_ (mGy) *	Small	1.4 ± 0.1	1.1 ± 0.03	0.7 ± 0.01	0.5 ± 0.01
	Medium	3.1 ± 0.04	2.3 ± 0.1	1.6 ± 0.1	0.9 ± 0.1
	Large	6.1 ± 0.2	4.7 ± 0.2	3.2 ± 0.1	1.6 ± 0.1

Values are the mean ± standard deviation (averaged for interscan-variability repetitions). * Calculated by the CT scanner. CTDI_Vol_ = volumetric computed tomography dose index.

**Table 2:** Comparison of image noise levels and contrast to noise ratios between TNC (70 keV, QIR off) and VNI (55 keV, QIR 1 and 60 keV, QIR 4) reconstructions.

Dose	Reconstruction	keV	QIR	IN	CNR
100%	TNC	70	off	16.7 ± 1.9	13.2 ± 1.3
	VNI	55	1	12.8 ± 1.7 *	7.5 ± 1.1 *
	VNI	60	4	7.7 ± 0.9 *^#^	10.6 ± 1.5 *^#^
75%	TNC	70	off	18.6 ± 2.1	11.7 ± 1.3
	VNI	55	1	14.6 ± 2.2 *	6.7 ± 0.8 *
	VNI	60	4	8.6 ± 1.2 *^#^	9.7 ± 1.2 *^#^
50%	TNC	70	off	22.0 ± 1.6	9.9 ± 0.7
	VNI	55	1	16.9 ± 1.8 *	6.2 ± 0.6 *
	VNI	60	4	10.1 ± 0.9 *^#^	8.5 ± 0.8 *^#^
25%	TNC	70	off	25.4 ± 2.0	8.7 ± 0.6
	VNI	55	1	20.9 ± 2.8 *	5.2 ± 0.9 *
	VNI	60	4	12.5 ± 1.8 *^#^	7.0 ± 1.6 *^#^

IN and CNR values are mean ± standard deviation. CNR = contrast to noise ratio; IN = image noise; QIR = quantum iterative reconstruction; TNC = true noncontrast; VNI = virtual noniodine. * Significantly different (*p* < 0.001) from TNC. ^#^ Significantly different (*p* < 0.001) from the other VNI technique.

**Table 3:** Comparison of Agatston scores between CACS_VNI_ at different radiation dose levels and CACS_TNC_ at standard radiation dose (100%).

	Dose	Total AS ^1^	r *	ICC *	Bias *	LoA *	CV (RSME) *^,#^
**TNC reconstruction**							
70 keV QIR off	100%	625.8 ± 24.4					
**VNI reconstructions**							
55 keV QIR 1	100%	625.5 ± 47.4	0.88	0.96	−0.04	−53.4/53.3	6.9 (1.6–9.6)
	75%	625.8 ± 35.7	0.88	0.96	0.01	−54.8/54.8	5.2 (2.9–6.8)
	50%	597.4 ± 39.6	0.88	0.96	−4.7	−55.3/46.1	6.6 (3.7–8.5)
	25%	599.0 ± 58.4	0.89	0.97	−4.5	−55.1/46.2	7.8 (4.8–10.0)
60 keV QIR 4	100%	618.1 ± 35.2	0.88	0.97	−1.3	−52.5/50.0	5.4 (2.6–7.2)
	75%	625.5 ± 26.0	0.88	0.96	−0.04	−55.6/55.5	4.0 (2.2–5.2)
	50%	602.2 ± 38.2	0.88	0.96	−3.9	−56.2/48.3	6.2 (2.8–8.3)
	25%	611.5 ± 57.3	0.89	0.96	−2.4	−57.2/52.4	7.1 (3.3–9.5)

AS values are mean ± standard deviation. * Comparison between CACS_VNI_ at different dose levels and CACS_TNC_ at standard dose (100%). ^#^ Values are % (95% confidence interval). AS = Agatston Score; CACS = coronary artery calcium scoring; CV = coefficient of variation; ICC = intraclass correlation; keV = kilo-electronvolt; LoA = limits of agreement; QIR = quantum iterative reconstruction; RSME = root-mean squared error; TNC = true noncontrast; VNI = virtual noniodine.

## Data Availability

The analyzed datasets during the current study are available from the corresponding author on reasonable request.

## Abbreviations

ACC	American College of Cardiology
AHA	America
AS	Agatston score
Bpm	Beats per minute
CACS	Coronary artery calcium scoring
CAD-RADS	Coronary Artery Disease-Reporting and Data System
CCTA	Coronary CT angiography
CNR	Contrast-to-noise ratio
CT	Computed tomography
CTDI_Vol_	Volumetric computed tomography dose indices
CV	Coefficient of variation
ECG	Electrocardiogram
HU	Hounsfield units
ICC	Intraclass correlation coefficient
IN	Image noise
IR	Iterative reconstruction
LoA	Limits of agreement
ns	Not significant
PCD	Photon-counting detector
QIR	Quantum iterative reconstruction
RMSE	Root-mean squared error
ROI	Region of interest
SD	Standard deviation
TNC	True noncontrast
VMI	Virtual monoenergetic image
VNC	Virtual noncontrast
VNI	Virtual noniodine

## References

[1] Grandhi G.R., Mirbolouk M., Dardari Z.A., Al-Mallah M.H., Rumberger J.A., Shaw L.J., Blankstein R., Miedema M.D., Berman D.S., Budoff M.J. (2020). Interplay of Coronary Artery Calcium and Risk Factors for Predicting CVD/CHD Mortality: The CAC Consortium. JACC Cardiovasc. Imaging.

[2] Divakaran S., Cheezum M.K., Hulten E.A., Bittencourt M.S., Silverman M.G., Nasir K., Blankstein R. (2015). Use of Cardiac CT and Calcium Scoring for Detecting Coronary Plaque: Implications on Prognosis and Patient Management. Br. J. Radiol..

[3] Polonsky T.S., McClelland R.L., Jorgensen N.W., Bild D.E., Burke G.L., Guerci A.D., Greenland P. (2010). Coronary Artery Calcium Score and Risk Classification for Coronary Heart Disease Prediction. JAMA.

[4] Detrano R., Guerci A.D., Carr J.J., Bild D.E., Burke G., Folsom A.R., Liu K., Shea S., Szklo M., Bluemke D.A. (2008). Coronary Calcium as a Predictor of Coronary Events in Four Racial or Ethnic Groups. N. Engl. J. Med..

[5] Vakil P., Wen Z., Lima A.S., Weber E.J., Kallianos K.G., Elicker B.M., Naeger D.M., Henry T.S., Ordovas K.G. (2022). Predictive Value of Coronary Artery Calcium in Patients Receiving Computed Tomography Pulmonary Angiography for Suspected Pulmonary Embolism in the Emergency Department. J. Thorac. Imaging.

[6] Xia C., Vonder M., Sidorenkov G., Den Dekker M., Oudkerk M., van Bolhuis J.N., Pelgrim G.J., Rook M., de Bock G.H., van der Harst P. (2021). Cardiovascular Risk Factors and Coronary Calcification in a Middle-Aged Dutch Population: The ImaLife Study. J. Thorac. Imaging.

[7] Rumberger J.A., Brundage B.H., Rader D.J., Kondos G. (1999). Electron Beam Computed Tomographic Coronary Calcium Scanning: A Review and Guidelines for Use in Asymptomatic Persons. Mayo Clin. Proc..

[8] Agatston A.S., Janowitz W.R., Hildner F.J., Zusmer N.R., Viamonte M., Detrano R. (1990). Quantification of Coronary Artery Calcium Using Ultrafast Computed Tomography. J. Am. Coll. Cardiol..

[9] Arnett Donna K., Blumenthal Roger S., Albert Michelle A., Buroker Andrew B., Goldberger Zachary D., Hahn Ellen J., Himmelfarb Cheryl D., Amit K., Donald L.-J., William M.J. (2019). 2019 ACC/AHA Guideline on the Primary Prevention of Cardiovascular Disease. J. Am. Coll. Cardiol..

[10] Willemink M.J., Persson M., Pourmorteza A., Pelc N.J., Fleischmann D. (2018). Photon-Counting CT: Technical Principles and Clinical Prospects. Radiology.

[11] Kreisler B. (2022). Photon Counting Detectors: Concept, Technical Challenges, and Clinical Outlook. Eur. J. Radiol..

[12] Sandfort V., Persson M., Pourmorteza A., Noël P.B., Fleischmann D., Willemink M.J. (2021). Spectral Photon-Counting CT in Cardiovascular Imaging. J. Cardiovasc. Comput. Tomogr..

[13] Farhadi F., Rajagopal J.R., Nikpanah M., Sahbaee P., Malayeri A.A., Pritchard W.F., Samei E., Jones E.C., Chen M.Y. (2021). Review of Technical Advancements and Clinical Applications of Photon-Counting Computed Tomography in Imaging of the Thorax. J. Thorac. Imaging.

[14] Leng S., Yu Z., Halaweish A., Kappler S., Hahn K., Henning A., Li Z., Lane J., Levin D.L., Jorgensen S. (2016). Dose-Efficient Ultrahigh-Resolution Scan Mode Using a Photon Counting Detector Computed Tomography System. J. Med. Imaging.

[15] Sandstedt M., Marsh J., Rajendran K., Gong H., Tao S., Persson A., Leng S., McCollough C. (2021). Improved Coronary Calcification Quantification Using Photon-Counting-Detector CT: An Ex Vivo Study in Cadaveric Specimens. Eur. Radiol..

[16] Symons R., Pourmorteza A., Sandfort V., Ahlman M.A., Cropper T., Mallek M., Kappler S., Ulzheimer S., Mahesh M., Jones E.C. (2017). Feasibility of Dose-Reduced Chest CT with Photon-Counting Detectors: Initial Results in Humans. Radiology.

[17] Emrich T., Aquino G., Schoepf U.J., Braun F.M., Risch F., Bette S.J., Woznicki P., Decker J.A., O’Doherty J., Brandt V. (2022). Coronary Computed Tomography Angiography–Based Calcium Scoring: In Vitro and In Vivo Validation of a Novel Virtual Noniodine Reconstruction Algorithm on a Clinical, First-Generation Dual-Source Photon Counting-Detector System. Invest. Radiol..

[18] van Praagh G.D., Wang J., van der Werf N.R., Greuter M.J.W., Mastrodicasa D., Nieman K., van Hamersvelt R.W., Oostveen L.J., de Lange F., Slart R.H.J.A. (2022). Coronary Artery Calcium Scoring: Toward a New Standard. Investig. Radiol..

[19] Vingiani V., Abadia A.F., Schoepf U.J., Fischer A.M., Varga-Szemes A., Sahbaee P., Allmendinger T., Giovagnoli D.A., Hudson H.T., Marano R. (2020). Individualized Coronary Calcium Scoring at Any Tube Voltage Using a KV-Independent Reconstruction Algorithm. Eur. Radiol..

[20] Tesche C., De Cecco C.N., Vliegenthart R., Albrecht M.H., Varga-Szemes A., Duguay T.M., Ebersberger U., Bayer R.R., Canstein C., Schmidt B. (2017). Accuracy and Radiation Dose Reduction Using Low-Voltage Computed Tomography Coronary Artery Calcium Scoring with Tin Filtration. Am. J. Cardiol..

[21] Hecht H.S., de Siqueira M.E.M., Cham M., Yip R., Narula J., Henschke C., Yankelevitz D. (2015). Low-vs. Standard-Dose Coronary Artery Calcium Scanning. Eur. Heart J.—Cardiovasc. Imaging.

[22] Rutten A., Isgum I., Prokop M. (2008). Coronary Calcification: Effect of Small Variation of Scan Starting Position on Agatston, Volume, and Mass Scores. Radiology.

[23] Groen J., Greuter M., Vliegenthart R., Suess C., Schmidt B., Zijlstra F., Oudkerk M. (2008). Calcium Scoring Using 64-Slice MDCT, Dual Source CT and EBT: A Comparative Phantom Study. Int. J. Cardiovasc. Imaging.

[24] Renker M., Ramachandra A., Schoepf U.J., Raupach R., Apfaltrer P., Rowe G.W., Vogt S., Flohr T.G., Kerl J.M., Bauer R.W. (2011). Iterative Image Reconstruction Techniques: Applications for Cardiac CT. J. Cardiovasc. Comput. Tomogr..

[25] Sartoretti T., Landsmann A., Nakhostin D., Eberhard M., Roeren C., Mergen V., Higashigaito K., Raupach R., Alkadhi H., Euler A. (2022). Quantum Iterative Reconstruction for Abdominal Photon-Counting Detector CT Improves Image Quality. Radiology.

[26] Willemink M.J., den Harder A.M., Foppen W., Schilham A.M.R., Rienks R., Laufer E.M., Nieman K., de Jong P.A., Budde R.P.J., Nathoe H.M. (2016). Finding the Optimal Dose Reduction and Iterative Reconstruction Level for Coronary Calcium Scoring. J. Cardiovasc. Comput. Tomogr..

[27] Sartoretti T., Racine D., Mergen V., Jungblut L., Monnin P., Flohr T.G., Martini K., Frauenfelder T., Alkadhi H., Euler A. (2022). Quantum Iterative Reconstruction for Low-Dose Ultra-High-Resolution Photon-Counting Detector CT of the Lung. Diagnostics.

[28] Choi A.D., Leifer E.S., Yu J.H., Datta T., Bronson K.C., Rollison S.F., Schuzer J.L., Steveson C., Shanbhag S.M., Chen M.Y. (2019). Reduced Radiation Dose with Model Based Iterative Reconstruction Coronary Artery Calcium Scoring. Eur. J. Radiol..

[29] Racine D., Mergen V., Viry A., Eberhard M., Becce F., Rotzinger D.C., Alkadhi H., Euler A. (2023). Photon-Counting Detector CT With Quantum Iterative Reconstruction: Impact on Liver Lesion Detection and Radiation Dose Reduction. Investig. Radiol..

[30] Willemink M.J., Noël P.B. (2019). The Evolution of Image Reconstruction for CT—From Filtered Back Projection to Artificial Intelligence. Eur. Radiol..

[31] Fink N., Zsarnoczay E., Schoepf U.J., Griffith J.P., Wolf E.V., O’Doherty J., Suranyi P., Baruah D., Kabakus I.M., Ricke J. (2023). Photon Counting Detector CT-Based Virtual Non-Iodine Reconstruction Algorithm for In Vitro and In Vivo Coronary Artery Calcium Scoring—Impact of Virtual Monoenergetic and Quantum Iterative Reconstructions. Investig. Radiol..

[32] Zhang D., Scott A., Lee C., Gellada N., Hyun M., Zhou Y. (2021). Coronary Artery Calcium Scoring at Lower Tube Voltages—Dose Determination and Scoring Mechanism. Eur. J. Radiol..

[33] Voros S., Rivera J.J., Berman D.S., Blankstein R., Budoff M.J., Cury R.C., Desai M.Y., Dey D., Halliburton S.S., Hecht H.S. (2011). Guideline for Minimizing Radiation Exposure during Acquisition of Coronary Artery Calcium Scans with the Use of Multidetector Computed Tomography: A Report by the Society for Atherosclerosis Imaging and Prevention Tomographic Imaging and Prevention Councils in Collaboration with the Society of Cardiovascular Computed Tomography. J. Cardiovasc. Comput. Tomogr..

[34] Koo T.K., Li M.Y. (2016). A Guideline of Selecting and Reporting Intraclass Correlation Coefficients for Reliability Research. J. Chiropr. Med..

[35] van der Werf N.R., Booij R., Schmidt B., Flohr T.G., Leiner T., de Groen J.J., Bos D., Budde R.P.J., Willemink M.J., Greuter M.J.W. (2021). Evaluating a Calcium-Aware Kernel for CT CAC Scoring with Varying Surrounding Materials and Heart Rates: A Dynamic Phantom Study. Eur. Radiol..

[36] Nakazato R., Dey D., Gutstein A., Le Meunier L., Cheng V.Y., Pimentel R., Paz W., Hayes S.W., Thomson L.E.J., Friedman J.D. (2009). Coronary Artery Calcium Scoring Using a Reduced Tube Voltage and Radiation Dose Protocol with Dual-Source Computed Tomography. J. Cardiovasc. Comput. Tomogr..

[37] Dey D., Nakazato R., Pimentel R., Paz W., Hayes S.W., Friedman J.D., Cheng V.Y., Thomson L.E.J., Slomka P.J., Berman D.S. (2012). Low Radiation Coronary Calcium Scoring by Dual-Source CT with Tube Current Optimization Based on Patient Body Size. J. Cardiovasc. Comput. Tomogr..

[38] Tao S., Sheedy E., Bruesewitz M., Weber N., Williams K., Halaweish A., Schmidt B., Williamson E., McCollough C., Leng S. (2021). Technical Note: KV-Independent Coronary Calcium Scoring: A Phantom Evaluation of Score Accuracy and Potential Radiation Dose Reduction. Med. Phys..

[39] Apfaltrer G., Albrecht M.H., Schoepf U.J., Duguay T.M., De Cecco C.N., Nance J.W., De Santis D., Apfaltrer P., Eid M.H., Eason C.D. (2018). High-Pitch Low-Voltage CT Coronary Artery Calcium Scoring with Tin Filtration: Accuracy and Radiation Dose Reduction. Eur. Radiol..

[40] van der Werf N.R., van Gent M., Booij R., Bos D., van der Lugt A., Budde R.P.J., Greuter M.J.W., van Straten M. (2021). Dose Reduction in Coronary Artery Calcium Scoring Using Mono-Energetic Images from Reduced Tube Voltage Dual-Source Photon-Counting CT Data: A Dynamic Phantom Study. Diagnostics.

[41] Mergen V., Higashigaito K., Allmendinger T., Manka R., Euler A., Alkadhi H., Eberhard M. (2022). Tube Voltage-Independent Coronary Calcium Scoring on a First-Generation Dual-Source Photon-Counting CT—A Proof-of-Principle Phantom Study. Int. J. Cardiovasc. Imaging.

[42] van der Werf N.R., Greuter M.J.W., Booij R., van der Lugt A., Budde R.P.J., van Straten M. (2022). Coronary Calcium Scores on Dual-Source Photon-Counting Computed Tomography: An Adapted Agatston Methodology Aimed at Radiation Dose Reduction. Eur. Radiol..

[43] van der Werf N.R., Rodesch P.A., Si-Mohamed S., van Hamersvelt R.W., Greuter M.J.W., Leiner T., Boussel L., Willemink M.J., Douek P. (2022). Improved Coronary Calcium Detection and Quantification with Low-Dose Full Field-of-View Photon-Counting CT: A Phantom Study. Eur. Radiol..

[44] van Werkhoven J.M., Schuijf J.D., Gaemperli O., Jukema J.W., Kroft L.J., Boersma E., Pazhenkottil A., Valenta I., Pundziute G., de Roos A. (2009). Incremental Prognostic Value of Multi-Slice Computed Tomography Coronary Angiography over Coronary Artery Calcium Scoring in Patients with Suspected Coronary Artery Disease. Eur. Heart J..

[45] Leschka S., Scheffel H., Desbiolles L., Plass A., Gaemperli O., Stolzmann P., Genoni M., Luescher T., Marincek B., Kaufmann P. (2008). Combining Dual-Source Computed Tomography Coronary Angiography and Calcium Scoring: Added Value for the Assessment of Coronary Artery Disease. Heart.

[46] Cury Ricardo C., Jonathon L., Suhny A., Stephan A., Daniel B., Marcio B., Matthew B., Kavitha C., Andrew D., Brian G. (2022). CAD-RADSTM 2.0—2022 Coronary Artery Disease-Reporting and Data System. JACC Cardiovasc. Imaging.

[47] Gassert F.G., Schacky C.E., Müller-Leisse C., Gassert F.T., Pahn G., Laugwitz K.-L., Makowski M.R., Nadjiri J. (2021). Calcium Scoring Using Virtual Non-Contrast Images from a Dual-Layer Spectral Detector CT: Comparison to True Non-Contrast Data and Evaluation of Proportionality Factor in a Large Patient Collective. Eur. Radiol..

[48] van Osch J.A.C., Mouden M., van Dalen J.A., Timmer J.R., Reiffers S., Knollema S., Greuter M.J.W., Ottervanger J.P., Jager P.L. (2014). Influence of Iterative Image Reconstruction on CT-Based Calcium Score Measurements. Int. J. Cardiovasc. Imaging.

[49] Szilveszter B., Elzomor H., Károlyi M., Kolossváry M., Raaijmakers R., Benke K., Celeng C., Bartykowszki A., Bagyura Z., Lux Á. (2016). The Effect of Iterative Model Reconstruction on Coronary Artery Calcium Quantification. Int. J. Cardiovasc. Imaging.

[50] Tesche C., De Cecco C.N., Schoepf U.J., Duguay T.M., Albrecht M.H., Caruso D., Varga-Szemes A., Lesslie V.W., Ebersberger U., Canstein C. (2017). Iterative Beam-Hardening Correction with Advanced Modeled Iterative Reconstruction in Low Voltage CT Coronary Calcium Scoring with Tin Filtration: Impact on Coronary Artery Calcium Quantification and Image Quality. J. Cardiovasc. Comput. Tomogr..

[51] Willemink M.J., Takx R.A.P., de Jong P.A., Budde R.P.J., Bleys R.L.A.W., Das M., Wildberger J.E., Prokop M., Buls N., de Mey J. (2014). The Impact of CT Radiation Dose Reduction and Iterative Reconstruction Algorithms from Four Different Vendors on Coronary Calcium Scoring. Eur. Radiol..

[52] Eberhard M., Mergen V., Higashigaito K., Allmendinger T., Manka R., Flohr T., Schmidt B., Euler A., Alkadhi H. (2021). Coronary Calcium Scoring with First Generation Dual-Source Photon-Counting CT—First Evidence from Phantom and In-Vivo Scans. Diagnostics.

[53] Keelan P.C., Bielak L.F., Ashai K., Jamjoum L.S., Denktas A.E., Rumberger J.A., Sheedy I., Patrick F., Peyser P.A., Schwartz R.S. (2001). Long-Term Prognostic Value of Coronary Calcification Detected by Electron-Beam Computed Tomography in Patients Undergoing Coronary Angiography. Circulation.

[54] Becker A., Leber A., Becker C., Knez A. (2008). Predictive Value of Coronary Calcifications for Future Cardiac Events in Asymptomatic Individuals. Am. Heart J..

[55] Craiem D., Casciaro M., Pascaner A., Soulat G., Guilenea F., Sirieix M.-E., Simon A., Mousseaux E. (2020). Association of Calcium Density in the Thoracic Aorta with Risk Factors and Clinical Events. Eur. Radiol..

[56] Razavi A.C., van Assen M., De Cecco C.N., Dardari Z.A., Berman D.S., Budoff M.J., Miedema M.D., Nasir K., Rozanski A., Rumberger J.A. (2022). Discordance Between Coronary Artery Calcium Area and Density Predicts Long-Term Atherosclerotic Cardiovascular Disease Risk. JACC Cardiovasc. Imaging.

[57] Greenland P., Alpert J.S., Beller G.A., Benjamin E.J., Budoff M.J., Fayad Z.A., Foster E., Hlatky M.A., Hodgson J.M.C.B., Kushner F.G. (2010). 2010 ACCF/AHA Guideline for Assessment of Cardiovascular Risk in Asymptomatic Adults: A Report of the American College of Cardiology Foundation/American Heart Association Task Force on Practice Guidelines Developed in Collaboration with the American Society of Echocardiography, American Society of Nuclear Cardiology, Society of Atherosclerosis Imaging and Prevention, Society for Cardiovascular Angiography and Interventions, Society of Cardiovascular Computed Tomography, and Society for Cardiovascular Magnetic Resonance. J. Am. Coll. Cardiol..

[58] Goff D.C., Lloyd-Jones D.M., Bennett G., Coady S., D’Agostino R.B., Gibbons R., Greenland P., Lackland D.T., Levy D., O’Donnell C.J. (2014). 2013 ACC/AHA Guideline on the Assessment of Cardiovascular Risk. Circulation.

[59] Kim K.P., Einstein A.J., Berrington de González A. (2009). Coronary Artery Calcification Screening: Estimated Radiation Dose and Cancer Risk. Arch. Intern. Med..

[60] Gerber T.C., Carr J.J., Arai A.E., Dixon R.L., Ferrari V.A., Gomes A.S., Heller G.V., McCollough C.H., McNitt-Gray M.F., Mettler F.A. (2009). Ionizing Radiation in Cardiac Imaging. Circulation.

